# Treatment of hepatocellular carcinoma with portal vein tumor thrombus: advances and challenges

**DOI:** 10.18632/oncotarget.15411

**Published:** 2017-02-16

**Authors:** Jin-Fang Jiang, Yong-Cong Lao, Bao-Hong Yuan, Jun Yin, Xin Liu, Long Chen, Jian-Hong Zhong

**Affiliations:** ^1^ Department of Chemotherapy, Affiliated Tumor Hospital of Guangxi Medical University, Nanning, China; ^2^ Department of General Surgery, Yan’An Hospital Affiliated to Kunming Medical University, Kunming, China; ^3^ Department of Radiation Oncology, Sichuan Cancer Hospital & Institute, Sichuan Cancer Center, School of Medicine, University of Electronic Science and Technology of China, Chengdu, China; ^4^ Department of Radiology, Affiliated Tumor Hospital of Guangxi Medical University, Nanning, China; ^5^ Department of Hepatobiliary Surgery, Affiliated Tumor Hospital of Guangxi Medical University, Nanning, China

**Keywords:** hepatic resection, hepatocellular carcinoma, portal vein tumor thrombosis, transarterial chemoembolization

## Abstract

Portal vein tumor thrombus is a frequent, challenging complication in hepatocellular carcinoma. Hepatocellular carcinoma patients with portal vein tumor thrombus may show worse liver function, less treatment tolerance and worse prognosis than patients without portal vein tumor thrombus, and they may be at higher risk of comorbidity related to portal hypertension. Western and some Asian guidelines stratify hepatocellular carcinoma with portal vein tumor thrombus together with metastatic hepatocellular carcinoma and therefore recommend only palliative treatment with sorafenib or other systemic agents. In recent years, more treatment options have become available for hepatocellular carcinoma patients with portal vein tumor thrombus, and an evidence-based approach to optimizing disease management and treatment has become more widespread. Nevertheless, consensus policies for managing hepatocellular carcinoma with portal vein tumor thrombus have not been established. This comprehensive literature review, drawing primarily on studies published after 2010, examines currently available management options for patients with hepatocellular carcinoma and portal vein tumor thrombus.

## INTRODUCTION

Hepatocellular carcinoma (HCC) is the third most common cause of cancer-related deaths worldwide [1], leading to over 600, 000 deaths annually [1–2]. HCC shows a strong propensity to invade the liver vasculature. During so-called macrovascular invasion (MVI), tumor cells invade the main portal veins or their branches, hepatic veins or their branches, or the inferior vena cava in the liver [3–4]. Portal vein tumor thrombus (PVTT) is the most common form of MVI in HCC. Approximately 10 to 60% of HCC patients have PVTT at the time of diagnosis [5–6].

The prognosis of HCC patients is much poorer in the presence of PVTT; their overall survival is only 2-4 months with supportive care [7–8]. The worse prognosis of HCC patients with PVTT may reflect several factors, including larger tumors, more numerous tumors, poorer tumor grade, worse liver function and higher serum levels of alpha-fetoprotein. These factors likely conspire to explain the low liver function, tumor aggressiveness, low chemotherapy tolerance and high risk of complications related to portal hypertension that are often observed in HCC patients with PVTT [9].

PVTT is considered a contraindication for initial hepatic resection or transarterial chemoembolization (TACE) by many systems and associations, including the Barcelona Clinic Liver Cancer staging system [10], the European Association for the Study of Liver Disease [11], the American Association for the Study of Liver Disease [12] and the Asian Pacific Association for the Study of the Liver [13]. These guidelines recommend sorafenib for patients with PVTT [10–13]. However, selected HCC patients with PVTT can benefit from hepatic resection or TACE, leading the American Hepato-Pancreato-Biliary Association [14] and the Japan Society of Hepatology [15] to recommend the consideration of hepatic resection or TACE for such patients.

Recent decades have seen advances in the management of HCC with PVTT, which are reviewed here. These advances and perspectives for the future are based primarily on randomized controlled trials, comparative or cohort studies and case series (not case reports) published after 2010 and indexed in PubMed.

## CLINICAL FEATURES AND CLASSIFICATION OF PVTT

Like Barcelona Clinic Liver Cancer stage B HCC [16–17], HCC with PVTT comprises a heterogeneous family of conditions varying in clinical characteristics and prognosis. Since patients with different types of PVTT can show markedly different treatment outcomes, several efforts have been made to develop a unified classification of PVTT to allow precise, personalized therapy.

The first PVTT classification system was the General Rules for the Clinical and Pathological Study of Primary Liver Cancer, developed by the Liver Cancer Study Group of Japan [18, 19]. This classification is based on the clinical characteristics, imaging and pathology findings and surgical outcomes. It classifies PVTT macroscopically into five grades: Vp0, no tumor thrombus in the portal vein; Vp1, presence of a tumor thrombus distal to, but not within, the second-order branches of the portal vein; Vp2, presence of a tumor thrombus in the second-order branches of the portal vein; Vp3, presence of a tumor thrombus in the first-order branches of the portal vein; and Vp4, presence of a tumor thrombus in the main trunk of the portal vein or a portal vein branch contralateral to the primarily involved lobe (or both) [18–20]. Based on analysis of 20, 850 HCC patients in Japan in 2006 and 2007, the most frequent type of PVTT was Vp0, accounting for 87.1% of cases; in contrast, Vp1-Vp4 each accounted for 2.6-3.9% of cases [21]. Similar results were obtained from microscopic pathology findings of surgical or biopsy specimens. In contrast, 5-year overall survival varied to a much greater extent among the Vp classes, based on analysis of more than 25, 000 HCC patients in Japan who underwent hepatic resection between 1994 and 2005: Vp0, 59.0%; Vp1, 39.1%; Vp2, 23.3%; Vp3/4, 18.3% [22].

In 2006, Mei et al. [23] reported a second PVTT classification system, which divided PVTT into five grades from proximal to distal: type I, involving the first-order branch (left or right trunk of portal vein); type II, involving the first-order branch (left or right trunk of portal vein) and the main trunk of the portal vein; type III, involving the first-order branches (left and right trunks of portal vein) and the main trunk of the portal vein; type IV, involving type III and the superior mesenteric vein or splenic vein; and type V, involving any of the types I-IV as well as extrahepatic metastasis. Moreover, the classification system divides PVTTs into three pathology types based on degree of necrosis: proliferative, necrotic and organized.

In 2007, Cheng and coworkers [24] proposed a third PVTT classification system, which is accepted by many liver centers in China. This system defines four types of PVTT: type I, involving segmental branches or above; type II, involving the right or left portal vein; type III, involving the main portal vein; and type IV, involving the superior mesenteric vein. In a retrospective study of 441 HCC patients with PVTT who underwent partial hepatic resection with or without portal thrombectomy, the following frequencies were observed for the different types of PVTT: type I, 32.7%; type II, 42.9%; type III, 19.5%; and type IV, 5.0%. The corresponding 1-, 2-, and 3-year overall survival rates were 54.8, 33.9, and 26.7%; 36.4, 24.9, and 16.9%; 25.9, 12.9, and 3.7%; and 11.1, 0, and 0% (*P* < 0.001) [25].

These three PVTT classification systems consider hepatic resection to be a feasible treatment option for HCC patients with PVTT. Prognosis is determined by the extent of the PVTT and its proximity to the main, or even contralateral, portal vein [9]. In general, patients with minor portal vein involvement have better prognosis than those with major portal vein involvement. In addition, other PVTT classification system was also proposed [26].

## MONOTHERAPIES AND ASSOCIATED PROGNOSIS

### Hepatic resection

In the 1980s, hepatic resection was considered an option only for patients with a tumor thrombus in a first-order branch of the portal vein that did not involve the confluence of the left and right portal veins [27, 28]. During this time, only studies from Asia reported on the safety and efficacy of hepatic resection for HCC with PVTT. Some years later, the ability of hepatic resection to treat tumor thrombus extending to the main portal trunk was reported [29, 30], and hepatic resection with or without thrombectomy to treat PVTT began to spread and be refined. It is currently a widespread practice, especially in Asian liver centers [31].

Since then, a growing number of comparative or cohort studies, mostly from Asian countries, have reported hepatic resection to be safe and effective for selected patients with HCC and PVTT (Table 1) [32–56]. Among these studies, the median rate of postoperative complications was 26% (range, 3-42%) and median mortality was 4.1% (range, 0-23.7%). Median survival time was 25.4 months (range, 8-64), and median rates of 1-, 2-, and 3-year overall survival were 62, 52, and 41%. Despite the large number of studies documenting good post-resection outcomes for carefully selected HCC patients with PVTT, the suitability of the procedure for such patients remains controversial [57, 58], and it is not recommended by official guidelines in the West [10–13].

**Table 1:** Prognoses of patients with HCC and PVTT treated by hepatic resection

Study	Country/region	Enrollment period	Total patients	Postoperative complications, %	In-hospital mortality, %	Median survival, mo.	Overall survival, %		
							1 yr	2 yr	3 yr
Chang 2012 [32]	Taiwan	1991-2006	160	-	2.7	22	58	46	34
Chen 2012 [33]	China	2006-2008	88	19.3	4.5	9	31	18	15
Chok 2014 [34]	Hong Kong	1989-2010	88	*23*	3.4	9	46	33	23
Kojima 2015 [35]	Japan	2001-2010	66	-	-	28	73	48	40
Kokudo 2016 [36]	Japan	2000-2007	1058	3.7	-	34	75	59	49
Lee 2016 [37]	Korea	2000-2011	40	-	-	20	60	42	33
Li 2016 [38]	China	2010-2013	95	-	0	8	25	0	0
Liu 2014 [39]	Taiwan	2002-2012	247	-	2.8	64	84	76	71
Matono 2012 [40]	Japan	1985-2005	29	3.0	-	36	62	42	24
Peng 2012 [41]	China	2002-2007	201	4.0	0.5	20	42	20	14
Roayaie 2013 [42]	USA	1992-2010	165	-	7.3	13	52	31	22
Shi 2010 [43]	China	2001-2003	406	32.8	0.2	-	34	18	13
Tang 2013 [44]	China	2006-2008	186	36.0	23.7	10	40	20	14
Torzilli 2013 [46]	France, Italy, Japan, Argentina, USA	1990-2009	297	42.0	3	36	76	56	49
Wang 2013 [110]	China	2003-2008	68	-	0	33	55	-	-
Wei 2016 [47]	China	2012-2014	74	-	-	14	74	40	-
Xiao 2015 [49]	China	2001-2008	234	-	-	18	40	21	16
Ye 2014 [51]	China	2007-2009	338	-	-	15	49	37	19
Zhang 2014 [52]	China	2005- 2009	272	32	1.1	13	50	39	26
Zhang 2016 [53]	China	2005-2012	252	35	1.5	15	69	46	34
Zheng 2016 [54]	China	2000-2008	96	35.4	-	33	78	62	48
Zhong 2014 [55]	China	2000-2007	248	27.0	4.4	-	81	62	46
Zhou 2015 [56]	China	-	152	-	-	20	87	64	56

*Abbreviations*: “-”, data not reported

These results, together with systematic study of resection outcomes according to type of PVTT, argue for expanding official guidelines to recognize hepatic resection as a first-line option for selected patients with HCC and PVTT and preserved liver function. Analysis of 1021 patients in Japan with Vp3 or Vp4 PVTT who underwent resection showed a 5-year survival rate of 18.3% [22]. Systematic review of 24 studies involving 4, 389 HCC patients with MVI showed that hepatic resection was associated with median mortality of 2.7% (range, 0-24%) and median overall survival from 50% at 1 year to 18% at 5 years [59, 60]. A large retrospective study in Japan found that the median overall survival of 2, 093 HCC patients with PVTT was 2.87 years after hepatic resection, compared to only 1.10 years for 4, 381 HCC patients with PVTT after non-resection treatments (*P* < 0.001) [36]. However, hepatic resection showed no overall survival benefit for patients in whom PVTT affected the main trunk or contralateral branch (Vp3 or Vp4) [36].

The available evidence, then, suggests that resection can be considered as initial therapy for HCC patients with type I or II (Vp0-Vp3) PVTT and preserved liver function. Surgeons should consider hepatic resection when it is feasible, though they should be prepared for the fact that the procedure is technically demanding [61].

### TACE or transarterial chemotherapy

TACE is a standard treatment for patients with unresectable HCC, but it is officially contraindicated for HCC patients with PVTT involving the main trunk or a first-order left or right branch of the portal vein because of the potential risk of hepatic insufficiency resulting from post-TACE ischemia [10–13]. In 1997, Lee and coworkers [62] reported that TACE could be safely performed on HCC patients with main-trunk PVTT, though they did not observe a significant survival benefit. Since then, an increasing number of studies have explored the role of TACE and, less often, transarterial chemotherapy (TAC) to treat HCC with PVTT (Table 2) [39, 41, 51, 63–74]. Median survival time among these studies was 9 months (range, 4-16). Median overall survival rates were 48% at 1 year, 32% at 2 years, and 18% at 3 years. Unfortunately, most of these studies did not report complications or mortality. Meta-analysis involving eight controlled trials also found TACE had potential for incurring a survival benefit for advanced HCC with PVTT, even with main portal vein obstruction [75].

**Table 2:** Nonsurgical multimodality treatments in patients with HCC and PVTT

Study	Country/region	Enrollment period	Sample size	Classification of PVTT	Multimodality treatment	Outcomes
Giorgio 2016 [96]	Italy	2011-2014	49	Vp4	RFA plus sorafenib	1- and 3-year OS were 60 and 26%
Kang 2014 [106]	China	2004-2008	34	Vp3 or 4	Stereotactic body radiotherapy plus TACE	Response rate was 88%
Long 2016 [107]	China	2010-2014	60	Vp1, 2, or 3	Microwave ablation plus TACE	1- and 3-year OS were 48 and 23%
Nagai 2015 [108]	Japan	2002-2009	18	Vp3 or 4	Sorafenib plus TAC	1- and 3-year OS were 36 and 18%
Wang 2016 [109]	China	2002-2014	31	Vp1, 2	TACE plus sorafenib	Median survival time 12 months
			45	Vp3	TACE plus sorafenib	Median survival time 9 months
			54	Vp3	TACE plus radiotherapy	Median survival time 11 months
			37	Vp4	TACE plus sorafenib	Median survival time 7 months
			56	Vp4	TACE plus radiotherapy	Median survival time 9 months

RFA, radiofrequency ablation; PVTT, portal vein tumor thrombosis; TACE, transarterial chemoembolization; TAC, transarterial chemotherapy.

Several studies suggest that hepatic resection is safer and more effective than TACE/TAC for many HCC patients with PVTT [39, 41, 51]. In one study [39], overall survival rates at 1, 3 and 5 years were significantly better for 247 HCC patients with PVTT who underwent hepatic resection (85, 68, 61%) than for 181 who underwent TACE (60, 42, and 33%; *P* < 0.001). The survival benefit of resection remained significant even after using propensity score matching to eliminate baseline differences between the treatment groups. The same study found that patients receiving TACE were at 2-fold higher risk of mortality than those receiving hepatic resection. Another study [41] also found overall survival rates at 1, 3, and 5 years to be significantly higher after resection (42.0, 14.1, and 11.1%) than after TACE (37.8, 7.3, and 0.5%; *P* < 0.001). Subgroup analysis based on type of PVTT showed that this overall survival benefit was observed among patients with type I or II PVTT (*P* < 0.05), but not among those with type III or IV PVTT (Cheng et al [24] types). A third study [51] found that hepatic resection was associated with better overall survival than TACE for HCC patients with PVTT.

The available evidence, then, suggests that while TACE is an option for patients with HCC and PVTT, it may be more appropriate for those with type III or IV PVTT (Cheng et al [24] types). Patients with resectable HCC, type I or II PVTT and preserved liver function may derive greater survival benefit from hepatic resection.

### Radiotherapy

Just a few decades ago, conventional radiotherapy was not recommended for HCC patients, regardless of whether they also had PVTT, out of fear that an inability to localize radiotherapy precisely could damage the liver or even cause liver failure. In 1994, Chen and coworkers [76] published preliminary results showing that radiotherapy could be used safely on HCC patients with PVTT, although it did not seem to be effective [76].

Since 2000, a growing number of studies have applied radiotherapy to HCC patients with PVTT, in part reflecting advances in radiotherapy technology, such as the advent of three-dimensional conformal radiotherapy (3D-CRT), which is associated with low radiotoxicity. Other radiotherapy methods include proton beam therapy, intensity-modulated radiotherapy, and stereotactic radiotherapy. Using 3D-CRT to treat 47 patients with HCC and PVTT, Bae and coworkers [77] obtained a response rate of 40%, median survival time of 8 months, and 1-year survival rate of 15%. Also using 3D-CRT, Rim and coworkers [78] reported a partial response rate of 55.6%, stable disease rate of 31%, progressive disease rate of 6.7%, and complete remission rate of 6.7%. Several additional studies have also suggested that radiotherapy is safe for HCC patients with PVTT and can improve their overall survival [79–83]. Much stronger evidence for clinical efficacy of radiotherapy has come from a much larger, multicenter study involving 985 HCC patients with PVTT in the main trunk and/or first branch [84]. The PVTT response rate was 51.8%, and median overall survival time was 10.2 months. Therefore, modern radiotherapy should be an option for patients with unresectable HCC and PVTT.

### Radioembolization with yttrium-90

Radioembolization is a transarterial form of brachytherapy in which yttrium-90-loaded microspheres are injected intra-arterially and generate tumor-killing radiation internally. This is a relatively new therapy for treating HCC with PVTT. Among six comparative or cohort studies [85–90] published in 2015 and 2016, median survival time of HCC patients with PVTT after radioembolization with yttrium-90 was 8 months (range, 3-18). Median overall survival was 38% at 1 year, 26% at 2 years, and 14% at 3 years. A systematic review of 14 clinical studies and three abstracts involving 722 patients with HCC and PVTT [91] reported the following median outcomes: time to progression, 5.6 months; disease control rate, 74.3%; complete response rate, 3.2%; partial response rate, 16.5%; stable disease rate, 31.3%; and survival time, 9.7 months. Frequent toxic effects were fatigue, nausea/vomiting, and abdominal pain, few of which required medical intervention.

The available evidence, then, suggests that radioembolization with yttrium-90 may be safe and effective for treating HCC with PVTT [92]. However, this evidence comes entirely from retrospective or uncontrolled prospective studies. Evidence from large, randomized controlled trials is needed.

### Sorafenib

Sorafenib is a particularly strong example of where clinical practice does not reflect the bulk of available evidence. Following the success of sorafenib in managing advanced HCC in two clinical trials [93, 94], investigators began to explore its safety and efficacy in HCC patients with PVTT. The results consistently point to small or no clinical benefit, especially in comparison to other treatments. A study by Jeong and coworkers [95] reported median overall survival time of only 3.1 months among 30 HCC patients with Vp3 or Vp4 PVTT after sorafenib monotherapy. In a randomized controlled trial with 99 HCC patients with cirrhosis and PVTT, Giorgio and coworkers [96] found that overall survival rates at 1, 2 and 3 years were significantly higher among those receiving sorafenib and radiofrequency ablation (60, 35, 26%) than among those receiving only sorafenib (37, 0, 0%). In another study, median survival time of HCC patients with PVTT in the main trunk or the first branch was similar after sorafenib (4.3 months) or radiotherapy (5.9 months; *P* = 0.115) [97]. When propensity score-matched patients were compared, median survival time was found to be significantly longer after radiotherapy (10.9 vs. 4.8 months; *P* = 0.025). A study [98] comparing TAC with sorafenib to treat HCC patients with PVTT found that TAC led to a significantly higher disease control rate (*P* < 0.001) as well as significantly longer median overall survival time (7.1 vs. 5.5 months, *P* = 0.011). Another study [99] comparing the combination of sorafenib and TACE with sorafenib alone to treat HCC with main PVTT found a similar disease control rate in the two groups, as well as similar median overall survival (7.0 vs. 6.0 months, *P* = 0.544).

The available evidence, then, indicates that sorafenib monotherapy is inferior to other monotherapies or combination treatments. This leads us to question the wisdom of palliative sorafenib therapy for HCC patients with PVTT. The observed maximal survival benefit of fewer than 3 months [95–99] seems outweighed by the drug's prohibitive cost and risk of adverse effects [100, 101].

## COMBINATION THERAPIES AND ASSOCIATED PROGNOSIS

### Surgery-based multimodal treatment

For selected patients with HCC and PVTT, hepatic resection appears to provide better outcomes than TACE/TAC, radiotherapy, radioembolization with yttrium-90, sorafenib or non-surgical combination therapies. Nevertheless, long-term overall survival after hepatic resection alone remains unsatisfactory because of the high rate of tumor recurrence and correspondingly low rate of disease-free survival [59, 102]. As a result, liver centers in the East and West are increasingly turning to combination therapies involving surgery. The rationale is that hepatic resection can eliminate the original tumor nodule and PVTT, while the non-surgical therapies can reduce the risk of recurrence. Eliminating the PVTT improves liver function, helping patients tolerate the multiple therapies.

Additional evidence for the efficacy of surgery-based combination therapy comes from a study [38] comparing 45 HCC patients with main PVTT who underwent both neoadjuvant 3D-CRT and hepatic resection, with 50 patients who received hepatic resection alone. The combination approach was associated with significantly lower rates of HCC recurrence (hazard ratio [HR], 0.36) and HCC-related death (HR 0.32). Such combination therapy may also be effective with adjuvant TACE or TAC [103–105]. Future studies should further explore surgery-based multimodal therapy.

### Multimodal treatment without surgery

Combination therapies that do not involve surgery are essential for managing HCC, particularly HCC with PVTT. They can be less traumatic than surgical approaches and offer lower risk of mortality and more rapid recovery; on the other hand, they are only palliative. Several non-surgical multimodal treatments have been reported, such as sorafenib and radiofrequency ablation, sorafenib and TACE/TAC, radiotherapy and TACE, as well as TACE and microwave or ethanol ablation. The combination of TACE and radiotherapy is the most frequently used non-surgical multimodal treatment based on several studies (Table 2) [96, 106–109]. The relative efficacy of different combination therapies is difficult to assess because few studies have performed parallel comparisons, and comparisons across studies may be unreliable because of differences in patient characteristics.

## CONCLUSIONS

The available evidence suggests that hepatic resection may be appropriate first-line therapy for many HCC patients with Vp1-3 PVTT and preserved liver function, which would provide them access to a potentially curative treatment. In contrast, no curative treatment is currently available for HCC with Vp4 PVTT. Resection-based combination therapies may also be effective for many patients with HCC and PVTT, as long as preserved liver function is adequate. Future research is needed to optimize the type, dosing and timing of neoadjuvant or adjuvant treatments administered with hepatectomy in different HCC patients with PVTT. Future studies should focus on optimizing patient selection criteria for various combination therapies in order to maximize the benefits of resection. For patients with unresectable HCC and PVTT, then TACE/TAC, radiotherapy, or radioembolization with yttrium-90 should be considered. Future recommendations for managing HCC with PVTT must be based on clear evidence from large, well-designed, randomized controlled trials.

## Abbreviations

3D-CRT: three-dimensional conformal radiotherapy
HCC: hepatocellular carcinoma
MVI: macrovascular invasion
PVTT: portal vein tumor thrombosis
TACE: transarterial chemoembolization
